# The Remuneration of the Institutional Dispenser

**Published:** 1912-09-07

**Authors:** Frederic Bullen

**Affiliations:** 85 Lydhurst Avenue, Streatham Hill, S.W.


### The Remuneration of the Institutional Dispenser.

*To the Editor of* The Hospital.

Sir,—Further to your instructive article on "Dispensing Anomalies in Public Institutions," may I point out one of the factors which, in my opinion, tend to preserve if not to create such undesirable absurdities? I refer to the inadequate remuneration so frequently offered to the institution dispenser. Many of the large London hospitals offer only £100 per annum, a women and children's hospital in an eastern suburb pays £80 per annum, and quite recently a county mental hospital advertised for a dispenser to whom they offered £80 per annum. Had the secretary or medical superintendent required a junior clerk, whom they would have instructed in his duties, or a steward, whose training would have been the result of experience only, a similar salary would probably be offered, with greater possibilities of advancement. Where an officer is required whose training has been costly, whose certificates of qualification are already obtained, and who must have proved his efficiency before appointment, the salary is hopelessly insufficient.

It is only fair to suppose that there will be attracted to such posts a type of dispenser who is worth £80 per annum, and the whole of the craft must bear the ill-effects of this fact. During recent years brave attempts have been made to improve the quality of the institution dispenser; he is now becoming a specialist in his work, but committees and medical staffs must remember that the best men need an equitable return, and are, in the long run, worth it.—Faithfully yours,

Frederic Bullen, M.P.S.

85 Lydhurst Avenue, Streatham Hill, S.W.,
September 2.

